# Combined “*de novo*” and “*ex novo*” lipid fermentation in a mix-medium of corncob acid hydrolysate and soybean oil by *Trichosporon dermatis*

**DOI:** 10.1186/s13068-017-0835-8

**Published:** 2017-06-09

**Authors:** Chao Huang, Mu-Tan Luo, Xue-Fang Chen, Gao-Xiang Qi, Lian Xiong, Xiao-Qing Lin, Can Wang, Hai-Long Li, Xin-De Chen

**Affiliations:** 1 CAS Key Laboratory of Renewable Energy, Chinese Academy of Sciences, Guangzhou, 510640 People’s Republic of China; 2 Guangzhou Institute of Energy Conversion, Chinese Academy of Sciences, No.2 Nengyuan Road, Tianhe District, Guangzhou, 510640 People’s Republic of China; 3 Guangdong Provincial Key Laboratory of New and Renewable Energy Research and Development, Guangzhou, 510640 People’s Republic of China; 4 University of Chinese Academy of Sciences, Beijing, 100049 People’s Republic of China

**Keywords:** “*de novo*” and “*ex novo*” lipid fermentation, Corncob acid hydrolysate, Soybean oil, Microbial oil, *Trichosporon dermatis*

## Abstract

**Background:**

Microbial oil is one important bio-product for its important function in energy, chemical, and food industry. Finding suitable substrates is one key issue for its industrial application. Both hydrophilic and hydrophobic substrates can be utilized by oleaginous microorganisms with two different bio-pathways (“*de novo*” lipid fermentation and “*ex novo*” lipid fermentation). To date, most of the research on lipid fermentation has focused mainly on only one fermentation pathway and little work was carried out on both “*de novo*” and “*ex novo*” lipid fermentation simultaneously; thus, the advantages of both lipid fermentation cannot be fulfilled comprehensively.

**Results:**

In this study, corncob acid hydrolysate with soybean oil was used as a mix-medium for combined “*de novo*” and “*ex novo*” lipid fermentation by oleaginous yeast *Trichosporon dermatis*. Both hydrophilic and hydrophobic substrates (sugars and soybean oil) in the medium can be utilized simultaneously and efficiently by *T. dermatis*. Different fermentation modes were compared and the batch mode was the most suitable for the combined fermentation. The influence of soybean oil concentration, inoculum size, and initial pH on the lipid fermentation was evaluated and 20 g/L soybean oil, 5% inoculum size, and initial pH 6.0 were suitable for this bioprocess. By this technology, the lipid composition of extracellular hydrophobic substrate (soybean oil) can be modified. Although adding emulsifier showed little beneficial effect on lipid production, it can modify the intracellular lipid composition of *T. dermatis*.

**Conclusions:**

The present study proves the potential and possibility of combined “*de novo*” and “*ex novo*” lipid fermentation. This technology can use hydrophilic and hydrophobic sustainable bio-resources to generate lipid feedstock for the production of biodiesel or other lipid-based chemical compounds and to treat some special wastes such as oil-containing wastewater.

## Background

Single cell oil (SCO), namely microbial oil, is the lipid accumulated by oleaginous microorganisms in their cell body [1]. Because of its important function in energy, chemical, and food industry, it has been the focus of many researches [2, 3]. For large-scale production of SCO, finding suitable substrates is one important factor, and both hydrophilic and hydrophobic substrates can be utilized by oleaginous microorganisms for fermentation [2–4]. But the lipid fermentation on hydrophilic and hydrophobic substrates are carried out in two different bio-pathways: “*de novo*” lipid fermentation means the fermentation on hydrophilic substrates (sugars and related substrates), while “*ex novo*” lipid fermentation means the fermentation on hydrophobic substrates (oils, alkane, etc.) [3, 4]. The pathways of both “*de novo*” lipid fermentation and “*ex novo*” lipid fermentation have been elucidated clearly that the principal biochemical difference exists between “*de novo*” and “*ex novo*” lipid fermentation is that in the latter case, lipid accumulation happens simultaneously with cell growth, being entirely independent from nitrogen exhaustion from the culture medium; and generation of acetyl-CoA, the necessary unit for lipid biosynthesis in oleaginous yeast, is different between “*de novo*” and “*ex novo*” lipid fermentation [4, 5]. It is worth noting that both “*de novo*” and “*ex novo*” lipid fermentation have their advantages: “*de novo*” lipid fermentation usually exhibits great lipid production [2, 3], while “*ex novo*” lipid fermentation can modify the extracellular and intracellular lipid composition to satisfy the requirement of food or chemical industry [3, 6, 7].

To date, most of the research on lipid fermentation has focused mainly on only one fermentation pathway (“*de novo*” lipid fermentation or “*ex novo*” lipid fermentation), and little work was carried out on both “*de novo*” and “*ex novo*” lipid fermentation simultaneously [3]; thus, the advantages of both lipid fermentation cannot be fulfilled comprehensively. For “*de novo*” lipid fermentation, lignocellulosic hydrolysate is considered as one ideal substrate for industrial production of SCO due to its advantages such as low cost, great availability, and renewable characteristics [2, 8, 9]. For “*ex novo*” lipid fermentation, vegetable oils or waste oils are usually used because of their advantages such as great availability, high fermentability, or low cost [10–13]. If “*de novo*” lipid fermentation and “*ex novo*” lipid fermentation can be carried out simultaneously, the hydrophilic and hydrophobic bio-resources from agriculture, food, and other fields (lignocellulosic biomass, vegetable oils, waste oils, etc.) can be both utilized efficiently, and also this technology has the potential to treat some special wastes such as oil-containing wastewater. Unfortunately, only a few studies have focused on this point.

**Scheme 1:**
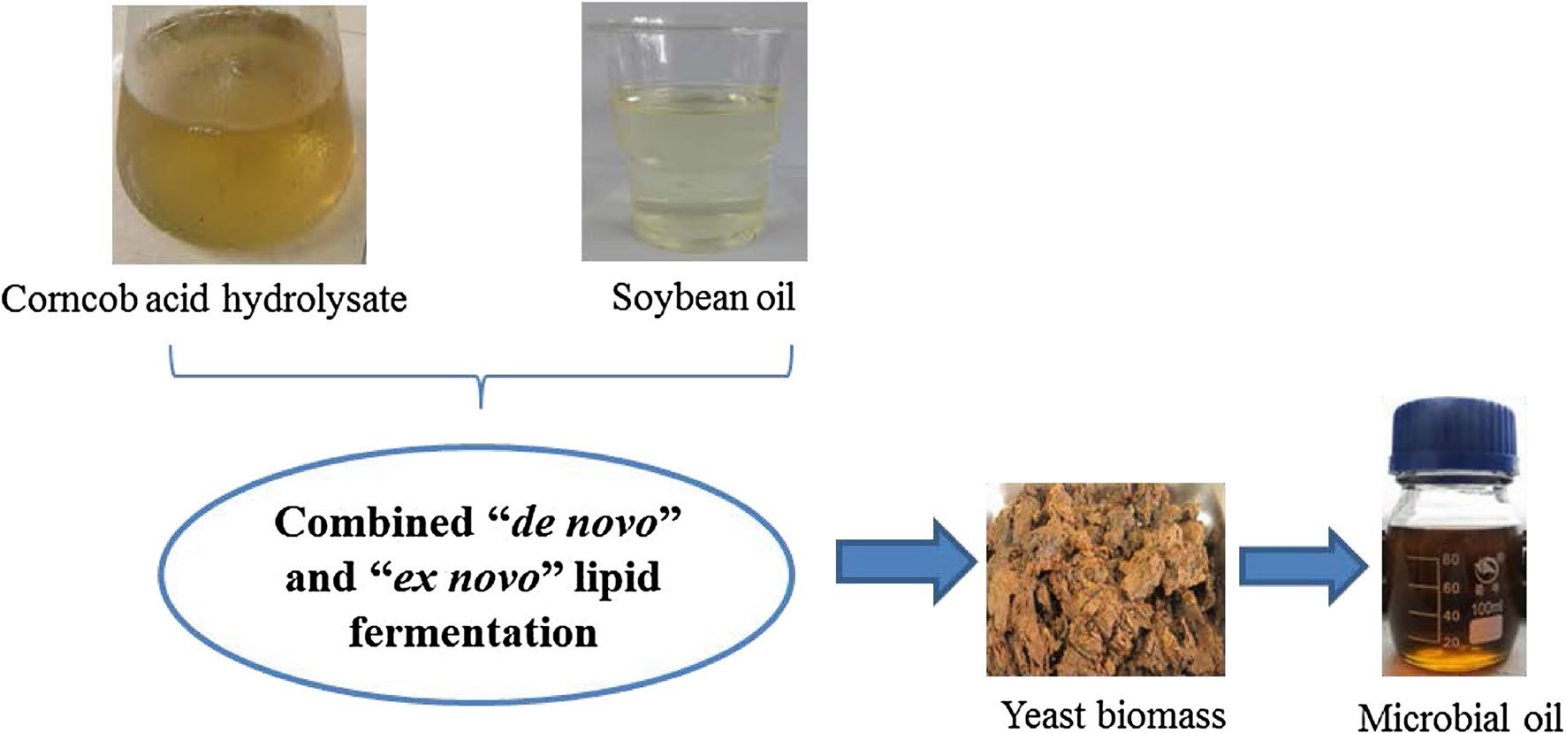
Combined “*de novo*” and “*ex novo*” lipid fermentation in a mix-medium of corncob acid hydrolysate and soybean oil

*Trichosporon dermatis* is one new kind of oleaginous yeast which has wide board of substrates, and especially it can utilize lignocellulosic hydrolysates (containing both hexose and pentose) efficiently for lipid fermentation [14–16]. If hydrophobic substrates can be utilized by this yeast, it can be one possible and potential microorganism for combined “*de novo*” and “*ex novo*” lipid fermentation. To evaluate the possibility and potential of combined “*de novo*” and “*ex novo*” lipid fermentation, the lipid fermentation by *T. dermatis* was carried out in the mix-medium containing both hydrophilic and hydrophobic substrates for the first time. To prevent potential inhibition on oleaginous yeast and its lipid fermentation, corncob acid hydrolysate and soybean oil were chosen as hydrophilic and hydrophobic substrates due to their high fermentability (Scheme 1). In detail, different fermentation modes were compared and the optimal mode was chosen firstly. Then, the effect of important fermentation factors (soybean oil concentration, inoculum size, and initial pH) on the lipid production was evaluated. Meanwhile, cell growth, lipid accumulation, and substrate utilization in the medium were measured throughout the fermentation. Finally, the effect of adding emulsifier on the lipid fermentation was analyzed. By this study, the system of combined “*de novo*” and “*ex novo*” lipid fermentation can be initially built.

## Methods

### Oleaginous microorganism and fermentation substrates

*Trichosporon dermatis* CH007 (Laboratory of Energy and Chemical Engineering, Guangzhou Institute of Energy Conversion, Chinese Academy of Sciences) was used for lipid fermentation in this work. Corncob acid hydrolysate was provided by ZHONGKE New Energy Co., Ltd (Ying-Kou, China). According to ZHONGKE New Energy Co., Ltd, the corncob acid hydrolysate has been detoxified by overliming and absorption by activated carbon. The reducing sugar concentration (measured by DNS method) of this substrate was 51.3 ± 0.2 g/L, and it mainly contained 2.9 g/L of glucose, 37.9 g/L of xylose, and 4.9 g/L of arabinose (measured by HPLC [17]). Soybean oil was obtained from local market (Guangdong, China), and its lipid composition (%) mainly included linoleic acid (53.8%), oleic acid (20.9%), palmitic acid (12.4%), linolenic acid (6.8%), and stearic acid (3.5%).

### Lipid fermentation

Corncob acid hydrolysate with soybean oil was used as the medium for combined “*de novo*” and “*ex novo*” lipid fermentation by *T. dermatis*. The yeast was cultured firstly on a medium (pH 6.0) containing (g/L) xylose, 20; peptone, 10; yeast extract, 10 at 28 °C and 150 rpm for 24 h. Then, certain seed culture was inoculated into the fermentation medium. Cultivation was performed in a 250-mL conical flask containing 50 mL of fermentation medium in a rotary shaker at 28 °C and 150 rpm.

### Comparison of different modes of combined “*de novo*” and “*ex novo*” lipid fermentation

Four fermentation modes including batch mode, fed-batch mode, first “*de novo*” then “*ex novo*” lipid fermentation, and first “*ex novo*” then “*de novo*” lipid fermentation were compared. For batch mode, soybean oil (20 g/L) was added into the corncob acid hydrolysate firstly before fermentation. Then the oleaginous yeast was added into the medium, and no extra substrate was added into the medium during fermentation. For fed-batch mode, the oleaginous yeast was added into the medium (corncob acid hydrolysate) firstly. Then, at the 48 and 96 h of fermentation, 10 and 10 g/L of soybean oil were added into the medium, respectively. For the mode of first “*de novo*” then “*ex novo*” lipid fermentation, the lipid fermentation was carried out in the corncob acid hydrolysate firstly for 3 days, and then 5% of culture in the corncob acid hydrolysate was translated into the medium (pure water) merely adding soybean oil (20 g/L) and the lipid fermentation in this medium was carried out for four days. For the mode of first “*ex novo*” then “*de novo*” lipid fermentation, the lipid fermentation was carried out in the medium (pure water) merely adding soybean oil (20 g/L) firstly for 3 days, and then 5% of culture in the medium merely containing soybean oil was translated into corncob acid hydrolysate and the lipid fermentation in this medium was carried out for 4 days. For comparison, a control experiment was also carried out in the control medium (corncob acid hydrolysate without adding soybean oil). The initial pH, inoculum size, fermentation temperature, and total fermentation period of all lipid fermentation modes were 6.0, 5%, 28 °C, and 7 days, respectively.

### Effect of soybean oil concentration, inoculum size, initial pH, and emulsifier addition on the combined “*de novo*” and “*ex novo*” lipid fermentation

To measure the effect of soybean oil concentration on lipid fermentation, five soybean oil concentrations (g/L, 0, 5, 10, 20, and 40) were used, and the inoculum size and initial pH were set at 5% and 6.0, respectively. To measure the effect of initial pH value on lipid fermentation, five different initial pH values (5.0, 6.0, 7.0, 8.0, and 9.0) were used, and the inoculum size and soybean oil concentration were set at 5% and 20 g/L, respectively. To study the effect of inoculum size on the lipid fermentation, five inoculum sizes (v/v, 1.0, 2.5, 5.0, 10.0, and 15.0%) were applied to culture, and the soybean oil concentration and initial pH were set at 20 g/L and 6.0, respectively. To study the effect of emulsifier on the lipid fermentation, four emulsifiers (Tween 80, Span 80, Tween 60, and OP-10) were added to culture with the concentration of 2 g/L, and the soybean oil concentration, inoculum size, and initial pH were set at 20 g/L, 5%, and 6.0, respectively. For comparison, a control experiment was also carried out in the control medium (corncob acid hydrolysate with adding 20 g/L soybean oil and without adding emulsifier). All the lipid fermentation was carried out for seven days at 28 °C.

### The courses of cell growth, lipid accumulation, and substrate utilization of combined “*de novo*” and “*ex novo*” lipid fermentation

The combined “*de novo*” and “*ex novo*” lipid fermentation was carried out in the corncob acid hydrolysate containing 20 g/L of soybean oil. The initial pH, inoculum size, and fermentation temperature of lipid fermentation were 6.0, 5%, and 28 °C, respectively. During lipid fermentation, the culture was withdrawn periodically (every 1 day) to evaluate the cell mass, lipid content, lipid production, residual sugars, and residual oils. After ten days of fermentation, the courses of cell growth, lipid accumulation, and substrate utilization of combined “*de novo*” and “*ex novo*” lipid fermentation were obtained.

### Analytical methods

Cell mass (g/L) was harvested by centrifugation at 8000*g* for 10 min and the dry cell weight was determined [18]. Extraction of intracellular lipid from dry cell mass was performed according to the modified procedure reported in a previous study [19], with a chloroform:methanol (2:1, v/v) mixture. The extracted intracellular lipid was recovered after the removal of solvent by a vacuum rotary evaporator. Lipid production is expressed as the amount of lipid extracted from the cells per liter fermentation broth (g/L) and lipid content is defined as the percentage of lipid to cell mass (%, w/w). Lipid yield (%, g/g) refers to the lipid production (g/L) on substrate (sugars and soybean oil) consumption (g/L). Residual sugar concentration was determined by DNS method [20]. The extracellular soybean oil (presented in the medium and attached to yeast cell mass) was recovered by hexane and the residual extracellular soybean oil concentration was determined by its dry weight after the removal of hexane by a vacuum rotary evaporator. After extraction by hexane, no soybean oil attached to yeast cell mass, and the remaining cell mass was used to determine the lipid content. The fatty acid composition was measured by converting fatty acids into fatty acid methyl esters and the fatty acid methyl esters were determined by gas chromatography (GC) (GC-7890, Agilent, USA) with an ionization detector and an HP-INNOWAX polyethylene glycol column (30 m × 250 μm × 0.25 μm). The column temperature was maintained at 170 °C for 1 min and then upgraded from 170 to 200 °C at a rate of 10 °C/min and kept for 1 min. After that, it was increased to 230 °C with a temperature gradient of 3 °C/min and held for 15 min. Argon was used as the carrier gas at 1.0 mL/min, with a split ratio of 1:10 (v/v). The injector temperature and detector temperature were both set at 240 °C. All the experiments were performed in duplicate and the results were expressed as the average.

## Results and discussion

### Comparison of different modes of combined “*de novo*” and “*ex novo*” lipid fermentation

**Fig. 1:**
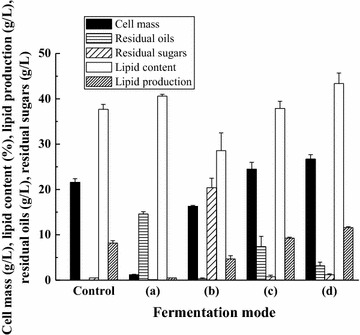
Effect of different fermentation modes on the combined “*de novo*” and “*ex novo*” lipid fermentation: *a* First “*de novo*” then “*ex novo*” lipid fermentation, *b* first “*ex novo*” then “*de novo*” lipid fermentation, *c* fed-batch mode, and *d* batch mode. For combined “*de novo*” and “*ex novo*” lipid fermentation (*a*–*d*), soybean oil was added into the corncob acid hydrolysate with different modes; for control experiment, fermentation was carried out in corncob acid hydrolysate without adding soybean oil

In this study, four fermentation modes including batch mode, fed-batch mode, first “*de novo*” then “*ex novo*” lipid fermentation, and first “*ex novo*” then “*de novo*” lipid fermentation (see details in “Methods”) were compared to evaluate their influence on the lipid fermentation of *T. dermatis* (Fig. 1). Generally, these four fermentation modes can be divided into two groups: batch mode and fed-batch mode can be classified as one-stage fermentation, while first “*de novo*” then “*ex novo*” lipid fermentation and first “*ex novo*” then “*de novo*” lipid fermentation can be classified as two-stage fermentation.

As shown in Fig. 1, for one-stage fermentation (batch mode and fed-batch mode), the cell mass and lipid content of *T. dermatis* were higher than those in the control medium (corncob acid hydrolysate without soybean oil). Compared with fed-batch mode, fermentation by *T. dermatis* with batch mode had higher cell mass and lipid content, and the utilization of sugars and soybean oil was more. In addition, batch mode is easy to set up and operate and thus more suitable for industrial applications [21]. First “*ex novo*” then “*de novo*” lipid fermentation showed lower cell mass and lipid content than that in the control medium, and also many sugars were still not consumed by *T. dermatis*. For the mode of first “*de novo*” then “*ex novo*” lipid fermentation, the cell mass of *T. dermatis* was extremely low, indicating that the existence of sugars in the fermentation medium was critical for combined “*de novo*” and “*ex novo*” lipid fermentation. Thus, the medium merely containing hydrophobic substrate might not be suitable for the lipid fermentation of *T. dermatis*. Different from the batch and fed-batch modes which are one-stage fermentation, the last two fermentation modes were two-stage fermentation and therefore they are difficult to be operated in the industry. Overall, the batch mode gave higher lipid production than the other modes.

The influence of different fermentation modes of combined “*de novo*” and “*ex novo*” lipid fermentation on the composition of intracellular lipid (microbial oil) and extracellular lipid (residual soybean oil) was further evaluated. As shown in Table 1, the lipid composition of microbial oil obtained from the batch and fed-batch modes of combined “*de novo*” and “*ex novo*” lipid fermentation was similar to that obtained from the control medium (corncob acid hydrolysate without soybean oil), showing that the influence of these two fermentation modes on the lipid composition of *T. dermatis* was little. In contrast, the fermentation modes of first “*de novo*” then “*ex novo*” lipid fermentation and first “*ex novo*” then “*de novo*” lipid fermentation showed more influence on the lipid composition of *T. dermatis* that the ratio of linoleic acid (C18:2) became lower and the ratios of stearic acid (C18:0) and palmitic acid (C16:0) were higher than that obtained from the control medium. Overall, combined “*de novo*” and “*ex novo*” lipid fermentation showed certain influence on the intracellular lipid composition, but this influence was not obvious.

**Table 1:** Effect of different fermentation modes on the intracellular lipid composition after combined “*de novo*” and “*ex novo*” lipid fermentation by *T. dermatis*

Fermentation modes	Lipid composition (%)						
	C16:0	C16:1	C18:0	C18:1	C18:2	C18:3	Others^a^
Control^b^	24.6 ± 0.6	0.5 ± 0.6	9.9 ± 0.4	40.4 ± 0.5	22.0 ± 0.1	1.1 ± 0.0	1.5 ± 0.2
First “*de novo*” then “*ex novo*” lipid fermentation	30.6 ± 0.4	0.7 ± 0.1	16.2 ± 0.0	44.0 ± 0.6	5.8 ± 1.3	0.2 ± 0.1	2.5 ± 0.2
First “*ex novo*” then “*de novo*” lipid fermentation	25.7 ± 0.0	0.6 ± 0.0	12.3 ± 0.1	41.9 ± 0.7	17.1 ± 0.6	0.9 ± 0.0	1.5 ± 0.0
Fed-batch mode	22.6 ± 1.1	0.4 ± 0.4	9.3 ± 0.7	38.3 ± 2.3	25.9 ± 3.5	2.0 ± 0.4	1.5 ± 0.2
Batch mode	23.6 ± 0.0	0.4 ± 0.5	9.2 ± 0.2	39.2 ± 1.6	24.5 ± 1.9	1.6 ± 0.3	1.5 ± 0.1

^a^Others were C12:0, C14:0, C20:0, C20:1, C20:2, and C24:0

^b^Fermentation in corncob acid hydrolysate without adding soybean oil

The influence of different fermentation modes on the extracellular lipid composition is shown in Table 2. As it is shown, the influence was more obvious on the extracellular lipid composition than on the intracellular lipid composition. For the batch mode of fermentation, the ratio of linoleic acid (C18:2) in extracellular lipid decreased greatly as the ratio of oleic acid (C18:1), stearic acid (C18:0), and palmitic acid (C16:0) increased. The influence of first “*ex novo*” then “*de novo*” lipid fermentation on the extracellular lipid composition was less than that of batch mode, followed by the effect of fed-batch mode, and the effect of first “*de novo*” then “*ex novo*” lipid fermentation was the least. Undoubtedly, although the combined “*de novo*” and “*ex novo*” lipid fermentation of *T. dermatis* showed an unobvious effect on the intracellular lipid composition, it can alter the extracellular lipid composition greatly and thus fulfill the in situ modification of soybean oil by lipid fermentation to satisfy the requirement of different industrial applications of lipid [3]. More specially, lipid with a lower degree of unsaturation may have some important functions in food [22] and is more suitable for the synthesis of chemical products such as special surfactant or fuel due to its higher oxidative stability [23, 24]. However, in the case of unsaturated lipid, it is more suitable for the synthesis of epoxide and its derivatives [25]. Thus, the in situ modification of oils can expand the application of combined “*de novo*” and “*ex novo*” lipid fermentation and increase its profits.

**Table 2:** Effect of different fermentation modes on the extracellular lipid composition after combined “*de novo*” and “*ex novo*” lipid fermentation by *T. dermatis*

Fermentation modes	Lipid composition (%)						
	C16:0	C16:1	C18:0	C18:1	C18:2	C18:3	Others^a^
Soybean oil (substrate)	12.4 ± 0.7	0.2 ± 0.0	3.5 ± 0.2	20.9 ± 1.1	53.8 ± 1.3	6.8 ± 0.2	2.4 ± 0.9
Control^b^	NA						
First “*de novo*” then “*ex novo*” lipid fermentation	12.6 ± 0.0	0.0 ± 0.0	4.9 ± 0.0	27.1 ± 0.1	53.5 ± 0.2	0.5 ± 0.0	1.3 ± 0.3
First “*ex novo*” then “*de novo*” lipid fermentation	23.2 ± 1.8	1.2 ± 1.7	12.3 ± 0.5	20.3 ± 10.2	37.6 ± 6.3	4.5 ± 1.1	0.9 ± 1.2
Fed-batch mode	14.5 ± 3.2	0.1 ± 0.1	5.9 ± 1.6	29.4 ± 4.0	47.7 ± 9.0	0.5 ± 0.1	1.9 ± 0.3
Batch mode	25.4 ± 0.2	0.1 ± 0.2	10.9 ± 0.4	44.5 ± 1.3	14.3 ± 0.3	0.5 ± 0.2	4.2 ± 0.5

*NA* not available

^a^Others were C12:0, C14:0, C20:0, C20:1, C20:2, and C24:0

^b^Fermentation in corncob acid hydrolysate without adding soybean oil

In summary, compared with fermentation in the control medium, the cell mass (21.6 g/L vs. 26.7 g/L), lipid content (37.7% vs. 43.4%), and lipid production (8.2 g/L vs. 11.6 g/L) of *T. dermatis* in the medium of combined “*de novo*” and “*ex novo*” lipid fermentation with batch mode were higher for about 23.6, 15.1, and 41.5%, respectively. Also, most hydrophilic and hydrophobic substrates were consumed by *T. dermatis* for the batch mode of combined “*de novo*” and “*ex novo*” lipid fermentation (about 97.7% of sugars and 84.0% of soybean oil were utilized). Although the effect of batch mode of combined “*de novo*” and “*ex novo*” lipid fermentation on the intracellular lipid composition was not obvious, it can modify the extracellular lipid composition greatly. Considering the above results, batch mode was chosen as the basic fermentation mode for later research on the combined “*de novo*” and “*ex novo*” lipid fermentation.

### Effect of soybean oil concentration, inoculum size, and initial pH on the combined “*de novo*” and “*ex novo*” lipid fermentation

**Fig. 2:**
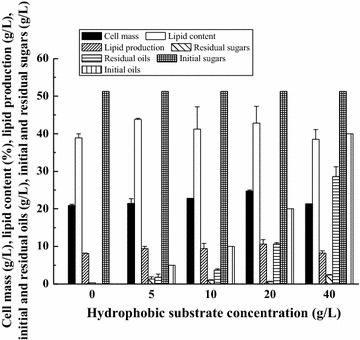
Effect of hydrophobic substrate (soybean oil) concentration on the combined “*de novo*” and “*ex novo*” lipid fermentation

In this study, the hydrophilic substrate used in combined “*de novo*” and “*ex novo*” lipid fermentation was corncob acid hydrolysate (its sugar concentration was fixed and it is suitable for lipid fermentation of *T. dermatis* [14]), and the typical hydrophobic substrate (soybean oil) was added into corncob acid hydrolysate to build the fermentation system. However, the effect of soybean oil concentration on the combined “*de novo*” and “*ex novo*” lipid fermentation was still unknown and this will be explained in this part. As shown in Fig. 2, the lipid production of *T. dermatis* was higher as the soybean oil concentration increased to 20 g/L, but the lipid production became lower when the soybean oil concentration was 40 g/L, indicating that some substrate inhibition existed when the soybean oil concentration was high. Overall, soybean oil concentration of 20 g/L was suitable for the combined “*de novo*” and “*ex novo*” lipid fermentation.

**Fig. 3:**
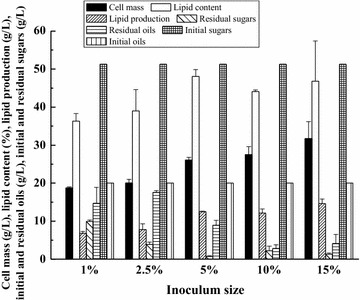
Effect of inoculum size on the combined “*de novo*” and “*ex novo*” lipid fermentation

For lipid fermentation in lignocellulosic hydrolysates, inoculum size and initial pH are the two factors that might affect the final lipid production [17]. In many studies, higher inoculum size will help microorganism to overcome the inhibition of inhibitors in lignocellulosic hydrolysates [26]. In this study, the effect of inoculum size from 1 to 15% on the combined “*de novo*” and “*ex novo*” lipid fermentation was evaluated. As shown in Fig. 3, as the inoculum size was higher within the range tested, the cell mass of *T. dermatis* increased gradually. However, the lipid content of *T. dermatis* became lower when the inoculum size was higher than 5%, indicating that more cell mass was converted into other intracellular products. The medium of seed culture was a synthetic medium containing sugars obtained from commercial sources; thus, the cost of pre-culture medium was much higher than that of fermentation medium (corncob acid hydrolysate). Although the inoculum size of 15% gave the highest lipid production of *T. dermatis*, the inoculum size of 5% seems a better choice for industrial application for saving the amount of seed culture to reduce the total fermentation cost.

**Fig. 4:**
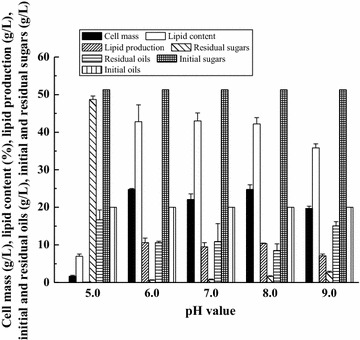
Effect of initial pH on the combined “*de novo*” and “*ex novo*” lipid fermentation

Besides the inoculum size, initial pH also has important influence on lipid fermentation [27, 28]. As shown in Fig. 4, when the initial pH ranged from 6.0 to 8.0, the lipid production of *T. dermatis* was similar, showing that *T. dermatis* has high pH adaptability in this pH range. When the initial pH was increased to 9.0, the cell mass and lipid content of *T. dermatis* became lower. Interestingly, when the initial pH was low (pH 5.0), *T. dermatis* even cannot grow, suggesting that acidic fermentation environment was not suitable for its combined “*de novo*” and “*ex novo*” lipid fermentation. Overall, the initial pH 6.0 gave the highest lipid production of *T. dermatis*.

### The courses of cell growth, lipid accumulation, and substrate utilization of combined “*de novo*” and “*ex novo*” lipid fermentation

**Fig. 5:**
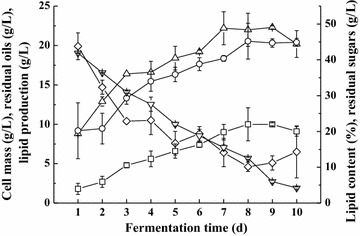
Cell growth, lipid accumulation, and substrate utilization during the combined “*de novo*” and “*ex novo*” lipid fermentation process; (*triangle*) cell mass; (*circle*) lipid content; (*square*) lipid production; (*inverted triangle*) residual sugars; (*diamond*) residual soybean oil

Using batch mode as the model fermentation system of combined “*de novo*” and “*ex novo*” lipid fermentation by *T. dermatis*, the cell growth, lipid accumulation, and substrate utilization during the fermentation process were further evaluated to learn the law of this bioconversion (Fig. 5). Interestingly, no lag phase was observed that the cell mass and lipid content of *T. dermatis* were 8.8 g/L and 20.2% respectively at the 1st day of fermentation, demonstrating that the corncob acid hydrolysate and soybean oil had slight toxicity to the cells of *T. dermatis*, which was also observed in another study merely using corncob acid hydrolysate as the substrate for lipid fermentation [14]. After that, the cell mass of *T. dermatis* increased gradually and the rate became slower from the 3rd day to the 7th day of fermentation. Then the cell mass was stable and became lower at the 10th day of fermentation. For lipid accumulation, the lipid content of *T. dermatis* increased in a relatively stable rate until the 8th day. But after that, the lipid content did not decrease, namely the phenomenon of “lipid turnover” (using intracellular lipid for maintaining the growth of oleaginous microorganism) [29–31] was not obvious for the combined “*de novo*” and “*ex novo*” lipid fermentation. Overall, the highest lipid production was obtained at the 8th day of fermentation, and this fermentation period was longer than that in the corncob hydrolysate without adding extracellular oils [14].

For combined “*de novo*” and “*ex novo*” lipid fermentation, both hydrophilic (sugars in corncob acid hydrolysate) and hydrophobic (soybean oil) substrates can be consumed by *T. dermatis* for cell growth and lipid accumulation (Fig. 5). The sugars were used quickly such that about 10 g/L of sugars were consumed by *T. dermatis* during the 1st day of fermentation and the residual sugar concentration was 41.9 g/L, again proving that no lag phase existed for the combined “*de novo*” and “*ex novo*” lipid fermentation. After that, the sugar consumption rate became slower such that about 5 g/L of sugars was utilized per day on average before the 5th day of fermentation. From the 6th day to the 10th day of fermentation, the sugars consumed per day on average (about 4 g/L) were further lower. Finally, the residual sugar concentration after ten days’ fermentation was 4.2 g/L. For the utilization of hydrophobic substrate (soybean oil) by *T. dermatis*, the condition was different. During the first day of fermentation, soybean oil was not consumed and about 20 g/L of extracellular oils was still present in the medium. From the 2nd to the 3rd day, the soybean oil was consumed simultaneously by *T. dermatis* as the sugar utilization. And during this period, the rate of oil consumption was even faster than that of sugars. However, after the 3rd day, the consumption of extracellular oils became much slower and the rate was unstable as well. Finally, the residual extracellular oil concentration in the medium was about 6.5 g/L after 10 days of fermentation. Considering the substrate utilization, the lipid production and lipid yield (lipid production on consumption of both sugars and extracellular oils) at the 8th day were 10.0 g/L and 18.5% (g/g) respectively, which were higher than those in the corncob acid hydrolysate without extracellular oils [14]. It is worth noting that the lipid yield on hydrophilic substrate (sugars) and hydrophobic substrate (oils) is different [3, 4], but the comprehensive lipid yield on both substrates (sugars and soybean oil) in this study is still attractive and suitable for microbial oil production.

### Effect of emulsifier on the combined “*de novo*” and “*ex novo*” lipid fermentation

**Fig. 6:**
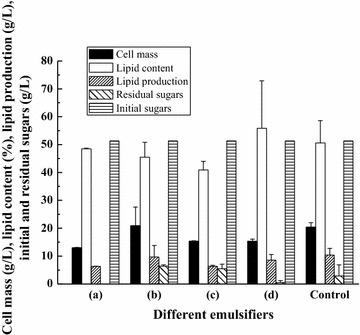
Effect of emulsifier on the combined “*de novo*” and “*ex novo*” lipid fermentation: *a* OP-10, *b* Tween 80, *c* Tween 60, and *d* Span 80. For fermentation (*a*–*d*), different emulsifiers were added into the media, respectively, while for control experiment fermentation was carried out in the medium without emulsifier

In this study, the fermentation system of combined “*de novo*” and “*ex novo*” lipid fermentation is special since it contained both hydrophilic substrate and hydrophobic substrate. Usually, for this system (water/oil biphasic system), adding emulsifier might help the utilization of different kinds of substrates especially for the hydrophobic substrates by different microorganisms [32]. Therefore, four emulsifiers including Tween 80, Span 80, Tween 60, and OP-10 were added into the system of combined “*de novo*” and “*ex novo*” lipid fermentation, respectively, to evaluate their effect on fermentation. As shown in Fig. 6, interestingly, adding emulsifiers into the fermentation system even showed a negative effect on the final lipid production. Moreover, adding emulsifiers made the recovery of extracellular hydrophobic substrates to be much more difficult because emulsifier and extracellular hydrophobic substrates might be both dissolved in the hydrophobic solvents for substrate recovery and thus the residual oil concentration in the medium was not measured in this part. Based on the above reason, it is not necessary to add emulsifiers in the actual application of combined “*de novo*” and “*ex novo*” lipid fermentation by *T. dermatis*. However, adding emulsifiers showed some effect on the intracellular lipid composition of microbial oil obtained from combined “*de novo*” and “*ex novo*” lipid fermentation. As shown in Table 3, adding Tween 80 could increase the ratio of linoleic acid (C18:2), and adding Span 80 and OP-10 could increase the ratio of oleic acid (C18:1). Overall, although adding emulsifiers showed little beneficial effect on the lipid production, its capacity to alter the intracellular lipid composition made it also one potential technology of industrial application of microbial oil in the food or other chemical industries [3].

**Table 3:** Effect of emulsifier on the intracellular lipid composition after combined “*de novo*” and “*ex novo*” lipid fermentation by *T. dermatis*

Emulsifier	Lipid composition (%)						
	C16:0	C16:1	C18:0	C18:1	C18:2	C18:3	Others^a^
Control (no emulsifier adding)	25.5 ± 2.4	1.6 ± 0.5	7.9 ± 0.2	37.3 ± 2.8	24.9 ± 0.6	1.6 ± 0.2	1.1 ± 0.3
Tween 80	21.5 ± 1.6	1.3 ± 0.2	6.5 ± 0.3	32.7 ± 1.3	34.3 ± 0.4	2.8 ± 0.1	0.9 ± 0.1
Span 80	27.1 ± 5.5	1.3 ± 0.1	8.3 ± 3.5	43.9 ± 3.0	16.8 ± 5.3	1.0 ± 0.7	1.5 ± 0.0
OP-10	24.0 ± 2.4	1.1 ± 0.4	11.7 ± 3.4	47.2 ± 4.3	14.5 ± 4.6	0.7 ± 0.2	0.9 ± 0.2
Tween 60	24.5 ± 0.2	1.1 ± 0.0	8.9 ± 0.5	38.6 ± 1.4	24.4 ± 1.5	1.5 ± 0.6	1.0 ± 0.1

^a^Others were C12:0, C14:0, C20:0, C20:1, C20:2, and C24:0

### Evaluation and outlook of the bioprocess

The main objective of this research is to evaluate the potential and possibility of combined “*de novo*” and “*ex novo*” lipid fermentation. To prevent potential inhibition on oleaginous yeast and its lipid fermentation, hydrophilic and hydrophobic substrates with high fermentability (corncob acid hydrolysate and soybean oil) were chosen to build the fermentation system. It is worth noting that little raw material contains both corncob acid hydrolysate and soybean oil in nature, and these two typical substrates are merely used as the model substrate in this study. For combined “*de novo*” and “*ex novo*” lipid fermentation, sugar (such as glucose, xylose, fructose, etc.) and fatty acid (oleic acid, stearic acid, palmitic acid, etc.) can be used as hydrophilic and hydrophobic substrates, respectively. Usually, it is difficult to find pure sugar or fatty acid in nature and pure sugar or fatty acid is seldom applied as a substrate in fermentation industry because the cost will be high. In the experiments, one typical hydrophilic substrate (corncob acid hydrolysate) and one typical hydrophobic substrate (soybean oil) were used simultaneously for lipid fermentation of oleaginous yeast. Compared with pure sugar and fatty acid, lignocellulosic biomass and vegetable oils are the natural materials that can be obtained or generated easily in nature, and therefore the potential and possibility of combined “*de novo*” and “*ex novo*” lipid fermentation in industrial application can be shown clearly using these materials as feedstock. However, after that the potential and possibility of combined “*de novo*” and “*ex novo*” lipid fermentation were proven by the present research, it is wise to evaluate the influence of different sugars and fatty acids on “*de novo*” and “*ex novo*” lipid fermentation in future and this will help in developing this technology.

Generally, the cost of agricultural residues such as straw, bagasse, and corncob is low and using lignocellulosic hydrolysates as the substrate for fermentation has great competitive advantages in industrial application [33]. For lignocellulosic biomass, corncob is usually used for xylitol production [34] and other lignocellulosic residues such as straw or bagasse might have more competitiveness for cost. Vegetable oils are usually used as food but the cost of waste vegetable oils from kitchen or industry is low. Therefore, future work should focus more on the fermentation on more low-cost hydrophilic and hydrophobic substrates such as waste lignocellulosic materials and waste oils for combined “*de novo*” and “*ex novo*” lipid fermentation especially for bioenergy purpose. Besides, two aspects will make this bioprocess more profitable in application. On the one hand, besides microbial oil, the residual yeast cell mass containing other valuable by-products such as cell wall polysaccharides can also make profits [35]. On the other hand, the residual hydrophobic substrate (e.g., soybean oil) can be modified that the saturation degree of oils can increase greatly, and this oil is more suitable for the synthesis of special food or other biochemical products [22–24] and makes more profits.

For biodiesel production, many forms of oils ranging from soybean oils to waste animal fats can be applied as feedstock [36], but many feedstock containing both hydrophilic and hydrophobic materials such as oil-containing wastewater cannot be simply converted into biofuels, and thus more exploitation on the application of these hydrophobic oil substrates is necessary. It is worth noting that most hydrophilic and hydrophobic substrates could be consumed after combined “*de novo*” and “*ex novo*” lipid fermentation (Fig. 1), and therefore this technology also has great potential for treatment of oil-containing wastewater. For treatment of oil-containing wastewater, adding hydrophilic substrates seems necessary because the efficiency of hydrophobic substrate utilization is low when the medium merely contains hydrophobic substrates (Fig. 1). In the actual application, lipid composition mainly decides the function and application of oil products and oils can be applied as biofuels, biochemical products, and health products with different lipid compositions [37–39]. The lipid composition of hydrophobic raw materials (vegetable oils or waste oils) varies greatly in nature [40], and thus future work should pay more attention on the influence of different raw materials on the composition of final oil products of combined “*de novo*” and “*ex novo*” lipid fermentation (both intracellular and extracellular lipids). Also, more hydrophobic materials with different initial lipid compositions and structures can be tried for combined “*de novo*” and “*ex novo*” lipid fermentation, and the modification on these hydrophobic substrates might offer suitable lipid composition for potential value-added applications.

Some basic parameters (soybean oil concentration, inoculum size, initial pH, and adding emulsifier) on lipid fermentation were initially evaluated to find out the possible factors that might affect the combined “*de novo*” and “*ex novo*” lipid fermentation, and future work should consider using more mathematical tools such as response surface analysis or orthogonal design to optimize the fermentation parameters especially when this technology is used for practical production. Last but not least, many aspects including cost of substrate, exploitation of fermentation by-products, and downstream technologies should be considered for industrial application of lipid fermentation [2], and even some details such as the amount of energy (aeration during fermentation, fermentation time, etc.) should be taken into account and compared to plant-based edible oil production cost. More work should be carried out to reduce the total cost of lipid fermentation and make this technology more competitive in industry.

## Conclusions

Overall, the combined “*de novo*” and “*ex novo*” lipid fermentation system containing both hydrophilic and hydrophobic substrates was successfully built. This study can offer one potential technology for utilization of sustainable bio-resources of agriculture, food, and other fields (lignocellulosic biomass, vegetable oils, waste oils, etc.) for lipid fermentation. Future study should focus on controlling the cost of this bioprocess using more low-cost materials as substrates, effect of different substrates on the product yield and composition, and more precise optimization of this bioprocess using mathematical tools in actual application.

## Abbreviations

SCO: single cell oil
HPLC: high-performance liquid chromatography
GC: gas chromatography
DNS: dinitrosalicylic acid

## References

[1] Ratledge C (2004). Fatty acid biosynthesis in microorganisms being used for single cell oil production. Biochimie.

[2] Huang C, Chen XF, Xiong L, Chen XD, Ma LL, Chen Y (2013). Single cell oil production from low-cost substrates: the possibility and potential of its industrialization. Biotechnol Adv.

[3] Papanikolaou S, Aggelis G (2011). Lipids of oleaginous yeasts. Part II: technology and potential applications. Eur J Lipid Sci Technol.

[4] Papanikolaou S, Aggelis G (2011). Lipids of oleaginous yeasts. Part I: biochemistry of single cell oil production. Eur J Lipid Sci Technol.

[5] Beopoulos A, Nicaud J-M, Gaillardin C (2011). An overview of lipid metabolism in yeasts and its impact on biotechnological processes. Appl Microbiol Biotechnol.

[6] Aggelis G, Papadiotis G, Komaitis M (1997). Microbial fatty acid specificity. Folia Microbiol.

[7] Papanikolaou S, Chevalot I, Komaitis M, Marc I, Aggelis G (2002). Single cell oil production by *Yarrowia lipolytica* growing on an industrial derivative of animal fat in batch cultures. Appl Microbiol Biotechnol.

[8] Jin M, Slininger PJ, Dien BS, Waghmode S, Moser BR, Orjuela A, Sousa LDC, Balan V (2015). Microbial lipid-based lignocellulosic biorefinery: feasibility and challenges. Trends Biotechnol.

[9] Sitepu IR, Garay LA, Sestric R, Levin D, Block DE, German JB, Boundy-Mills KL (2014). Oleaginous yeasts for biodiesel: current and future trends in biology and production. Biotechnol Adv.

[10] Aggelis G, Sourdis J (1997). Prediction of lipid accumulation-degradation in oleaginous micro-organisms growing on vegetable oils. Antonie Van Leeuwenhoek Int J Gen Mol Microbiol..

[11] Aggelis G, Komaitis M, Papanikolaou S, Papadopoulos G (1995). A mathematical model for the study of lipid accumulation in oleaginous microorganisms. II: study of cellular lipids of *Mucor circinelloides* during growth on a vegetable oil. Grasas y aceites.

[12] Papanikolaou S, Aggelis G (2003). Modeling lipid accumulation and degradation in *Yarrowia lipolytica* cultivated on industrial fats. Curr Microbiol.

[13] Papanikolaou S, Dimou A, Fakas S, Diamantopoulou P, Philippoussis A, Galiotou-Panayotou M, Aggelis G (2011). Biotechnological conversion of waste cooking olive oil into lipid-rich biomass using *Aspergillus* and *Penicillium* strains. J Appl Microbiol.

[14] Xiong L, Huang C, Yang XY, Lin XQ, Chen XF, Wang C, Wang B, Zeng XA, Chen XD (2015). Beneficial effect of corncob acid hydrolysate on the lipid production by oleaginous yeast *Trichosporon dermatis*. Prep Biochem Biotechnol.

[15] Huang C, Chen XF, Xiong L, Chen XD, Ma LL (2012). Oil production by the yeast *Trichosporon dermatis* cultured in enzymatic hydrolysates of corncobs. Bioresour Technol.

[16] Peng WF, Huang C, Chen XF, Xiong L, Chen XD, Chen Y, Ma LL (2013). Microbial conversion of wastewater from butanol fermentation to microbial oil by oleaginous yeast *Trichosporon dermatis*. Renew Energ..

[17] Chen XF, Huang C, Yang XY, Xiong L, Chen XD, Ma LL (2013). Evaluating the effect of medium composition and fermentation condition on the microbial oil production by *Trichosporon cutaneum* on corncob acid hydrolysate. Bioresour Technol.

[18] Xue FY, Miao JX, Zhang X, Luo H, Tan TW (2008). Studies on lipid production by *Rhodotorula glutinis* fermentation using monosodium glutamate wastewater as culture medium. Bioresour Technol.

[19] Folch J, Lees M, Sloane-Stanley G (1957). A simple method for the isolation and purification of total lipids from animal tissues. J Biol Chem.

[20] Miller G (1959). Use of dinitrosalicylic acid (DNS) for determination of reducing sugars. Anal Chem..

[21] Cinar A, Parulekar SJ, Undey C, Birol G (2003). Batch fermentation: modeling: monitoring, and control.

[22] Lipp M, Anklam E (1998). Review of cocoa butter and alternative fats for use in chocolate—part A. Compositional data. Food Chem.

[23] Falbe J (2012). Surfactants in consumer products: theory, technology and application.

[24] Knothe G (2005). Dependence of biodiesel fuel properties on the structure of fatty acid alkyl esters. Fuel Process Technol.

[25] Köckritz A, Martin A (2008). Oxidation of unsaturated fatty acid derivatives and vegetable oils. Eur J Lipid Sci Technol.

[26] Almeida J, Modig T, Petersson A, Hahn-Hagerdal B, Liden G, Gorwa-Grauslund M (2007). Increased tolerance and conversion of inhibitors in lignocellulosic hydrolysates by *Saccharomyces cerevisiae*. J Chem Technol Biotechnol.

[27] Johnson V, Singh M, Saini V, Sista V, Yadav N (1992). Effect of pH on lipid accumulation by an oleaginous yeast: *rhodotorula glutinis* IIP-30. World J Microbiol Biotechnol.

[28] Ageitos JM, Vallejo JA, Veiga-Crespo P, Villa TG (2011). Oily yeasts as oleaginous cell factories. Appl Microbiol Biotechnol.

[29] Fakas S, Galiotou-Panayotou M, Papanikolaou S, Komaitis M, Aggelis G (2007). Compositional shifts in lipid fractions during lipid turnover in *Cunninghamella echinulata*. Enzyme Microbial Technol..

[30] Holdsworth JE, Ratledge C (1988). Lipid turnover in oleaginous yeasts. J Gen Microbiol.

[31] Ratledge C, Wynn JP (2002). The biochemistry and molecular biology of lipid accumulation in oleaginous microorganisms. Adv Appl Microbiol.

[32] Garti N, Yaghmur A, Leser ME, Clement V, Watzke HJ (2001). Improved oil solubilization in oil/water food grade microemulsions in the presence of polyols and ethanol. J Agric Food Chem..

[33] Somerville C, Youngs H, Taylor C, Davis SC, Long SP (2010). Feedstocks for lignocellulosic biofuels. Science.

[34] Rivas B, Torre P, Domínguez JM, Converti A, Parajó JC (2006). Purification of xylitol obtained by fermentation of corncob hydrolysates. J Agric Food Chem..

[35] Qi GX, Huang C, Chen XF, Xiong L, Wang C, Lin XQ, Shi SL, Yang D, Chen XD (2016). Semi-pilot scale microbial oil production by *Trichosporon cutaneum* using medium containing corncob acid hydrolysate. Appl Biochem Biotechnol..

[36] Leung DYC, Wu X, Leung MKH (2010). A review on biodiesel production using catalyzed transesterification. Appl Energ.

[37] Osborn H, Akoh C (2002). Structured lipids-novel fats with medical, nutraceutical, and food applications. Compr Rev Food Sci Food Saf..

[38] Fox N, Stachowiak G (2007). Vegetable oil-based lubricants-a review of oxidation. Tribol Int.

[39] Gui MM, Lee K, Bhatia S (2008). Feasibility of edible oil vs. non-edible oil vs. waste edible oil as biodiesel feedstock. Energy.

[40] Gunstone F (2011). Vegetable oils in food technology: composition, properties and uses.

